# Gaming Behaviors among Polish Students with Visual Impairment

**DOI:** 10.3390/ijerph18041545

**Published:** 2021-02-06

**Authors:** Magdalena Agnieszka Wrzesińska, Klaudia Tabała, Patryk Stecz

**Affiliations:** 1 Department of Psychosocial Rehabilitation, Medical University of Lodz, 90-419 Lodz, Poland; klaudia.tabala@umed.lodz.pl; 2 Department of Clinical Psychology and Psychopathology, Faculty of Educational Sciences, Institute of Psychology, University of Lodz, 91-433 Lodz, Poland; patryk.stecz@now.uni.lodz.pl

**Keywords:** visual impairment, adolescents, young adults, digital games, behaviors, sociodemographic determinants

## Abstract

The access of people with disabilities to digital solutions promotes their inclusion and participation in many aspects of life. Computer games based on hearing or haptic devices have been gaining popularity among persons with visual impairment (VI), and players tend to display improved spatial and abstract reasoning skills, as well as better social interaction and self-confidence, after playing these games. However, a recent survey suggested that excessive gaming could represent a public health concern as a harmful form of behavior in young people associated with risk factors of negative psychosomatic and physical complaints. Young persons with VI are regular users of various technologies, but little is still known about their media patterns. This study aimed to determine the characteristics of the variables associated with gaming for adolescents with VI. The participants were 490 students, aged 13–24 years, from special schools for students with VI. Data was collected using a self-administered questionnaire. The current survey indicated a tendency towards excessive gaming in a significant proportion of young persons with VI. Sociodemographic variables are important in predicting gaming prevalence or screen time, but further research focused on establishing possible mediators (such as parental attitudes towards media) are necessary for identifying problematic gaming behaviors among students with VI.

## 1. Introduction

Digital games (DGs) have grown in popularity and represent a central component of youth culture today. In addition, they can also serve as effective educational tools that help young persons to think critically, develop intrinsic motivation, build collaborative skills, and increase cognitive performance [1,2,3,4]. Moreover, DGs provide an opportunity to relax, have fun, and avoid high-pressure and stressful environments [5,6].

Media use requires a wide range of behavioral actions depending on the hardware, device, formal features, or user interface [7]. The development of computer games also includes the ergonomic characteristics of the target population, including usability, playability, and effectiveness. Usability is related to functionality within the game system and the overall experience of a player, which can affect the interaction between the users and the game. On the other hand, playability concerns a broader sense of the system’s functionality, allowing for a successful and enjoyable interaction with a game. Effectiveness is the measure of a game’s ability to enhance knowledge acquisition and ability development in every user [8].

However, a recent survey suggested that excessive gaming could represent a public health concern as a harmful form of behavior in young people [9], which was also found to be associated with risk factors of negative psychosomatic and physical complaints [10,11].

Young persons, particularly adolescents, are vulnerable to technological addiction because of lower self-control, problems with long-term planning, and the inability to minimize the risk of dangerous behaviors or lower social competencies [12,13]. The prevalence of problematic DGs use has been reported to range from 1.7% to over 10% in general samples of young people [14,15]. Problematic gaming is also associated with lower levels of sociability, less perceived social support, lower expectations of self-efficacy, and lower life satisfaction [6]. Playing for more than 5 hours daily has also been found to elicit negative effects that manifest as problematic digital gaming, cyberbullying, or aggressive behaviors [16].

The online activity of young persons has been associated with certain sociodemographic variables. Male players are known to play more frequently, more intensively, and for longer amounts of time than female players [17], and urban adolescents are more likely to be computer users than those in rural areas [11]. In addition, it has been found that parental attitudes towards computer use and their own online activity affect the screen time of their children [18]. Problematic DGs use was observed more frequently within families experiencing domestic disputes than in those characterized by healthy family relationships [4].

Young persons with visual impairment (VI) are interested in a wide range of leisure activities, are regular users of computers and the internet, and tend to play DGs even more often than their sighted peers [19,20,21]. It has also been reported that in their leisure time, 60% of students with VI enjoy playing games on the computer and 33% like playing on their mobile phone [22]. A positive aspect of playing DGs is that it increases the potential for social inclusion among young persons with VI [23]. Studies have also shown that contact with new media is a factor compensating for social isolation faced by this group [19]. Hence, giving young persons with VI access to DGs promotes their inclusion and participation in society, thus also providing them equal access to youth culture [24]. 

Computer games based on hearing or haptic devices have been gaining popularity among persons with VI [25], and there is considerable interest in the use of computer games as educational and therapeutic instruments [25,26]. It is possible for this group to obtain cognitive benefits from playing DGs by using nonvisual forms of gaming, employing other sensory channels such as hearing, touch, or proprioception to gather relevant information. It has been shown that young persons with VI who play DGs tend to display improved special and abstract reasoning skills, as well as better social interaction and self-confidence [26].

In past decades, the popularity of information technologies and computer games has resulted in growing awareness of gaming’s potential benefits among researchers interested in learning and cognition [27]. It should be also mentioned that global improvements in the public’s access to technology provides an opportunity to develop acceptable and effective technologies in response to the needs of children and adolescents with long-term physical conditions. A new field of healthcare based on digital solutions, such as computer or smart phones and multimedia, interactive programs, serious games, or virtual reality, is being created. Moreover, it should be added that serious games today support health intervention in many health conditions, and the evidence of this intervention’s effectiveness is promising [28]. However, little is still known about new media patterns among young people with disabilities, and there is insufficient information describing their prevalence in this area [16]. Hence, it is necessary to know and understand the features of digital solutions selected by young people with disabilities, not only to improve the commercial development of games but also to adjust future e-health intervention to the interest of young people with disabilities. This study is a starting point for considering whether adolescents with VI are involved in playing computer games and to characterize selected variables related to digital gaming. Therefore, the aim of this study was to identify the time devoted to gaming sessions, the electronic devices used for digital gaming, and the prevalence of particular types of games played by students with VI, as well as the sociodemographic variables associated with gaming and the presence of excessive gaming (classified as 5 or more hours per day) in this population.

## 2. Methods

### 2.1. Participants

A total of 1018 students with VI were approached to take part in this study. All participants were students at nine randomly-selected Polish special schools for the blind and partially-sighted: Łódź, Wrocław, Kraków, Dąbrowa Górnicza, Bydgoszcz, Chorzów, Warszawa, Lublin, and Owińska. At the selection stage, no school declined participation in the survey. In total, 504 students met the survey criteria. The inclusion criteria were confirmation of VI in the school records based on ICD-10 [29], presence on the day of data collection, and the formal provision of informed consent. Exclusion criterions were intellectual disability and physical disabilities other than VI. Due to various factors such as withdrawal from research during implementation, lack of data, and illegible completion of the survey, 490 final respondents with VI between 13–24 years old (17.9 ± 2.48) were included in the study.

### 2.2. Procedure

A self-administered questionnaire assessing variables regarding gaming behaviors adapted for blind and partially-sighted students in Braille or large font was prepared for the present study. This cross-sectional study was conducted before the pandemic and data were collected from 2016 to 2017. The questionnaire recorded the mean time of gaming sessions, including time (in minutes) spent on gaming during school days and weekends based on the subjective student’s declaration. Excessive gaming was defined as more than 5 hours of play per day [30]. In the questionnaire, students also indicated the type of computer games they played. The study included the following types of games: strategy, fighting, sports, education/puzzles, action/adventure, war, and role-playing game simulation. The types of games included were based on a survey conducted among schoolchildren [31]. This survey, developed by Stránska et al., served as a source for assuring content validity of the measured constructs. The item analysis results suggest that the reliability of the survey instrument is adequate (Cronbach’s α = 0.794) and that the questions refer to a single construct related to the types of games played by each student. Sociodemographic variables were also collected and are shown in Table 1. The questionnaires were completed under the guidance of a trained team, comprised of a public health specialist and a psychologist.

### 2.3. Statistical Analysis

The chi-square test was used to assess the differences between subgroups with regards to electronic devices and specific game types. As the mean length of gaming followed a non-normal distribution, the nonparametric Mann–Whitney U-test was used to compare gender groups with regard to mean time devoted to game sessions, including sessions on school days and weekends. Gaming sessions lasting 5 or more hours included time spent playing during school days and weekends. Multivariate logistic regression was used to evaluate the association of age, type of VI, and sociodemographic variables with excessive gaming lasting 5 or more hours per day [26]. Confidence intervals (CIs) were presented as 95% CI. For all analyses, a *p*-value under 0.05 was considered to be significant. Participants who reported never playing games were excluded from in-depth statistical analyses.

## 3. Results

### 3.1. Time Devoted to Gaming and Choice of Electronic Devices

Almost 84% (410) of students with VI had experience in playing DGs. The sociodemographic characteristic of the gamers are presented in Table 1. Most of the poor-sighted students in the study had experience with gaming and were significantly more likely to be players than those who were blind (*n* = 365; 89% (poor-sighted) vs. *n* = 45; 11% (blind) χ^2^ = 22.471; *p* < 0.001). More male (57.1%) than female students (42.9%) with VI reported playing DGs (χ^2^ = 17.913; *p* < 0.001). Age was found to have no significant impact on gaming prevalence. The students with VI from urban areas were twice as likely to play games as those living in rural areas (χ ^2^ = 4.295; *p* = 0.038). A slight, insignificant tendency was observed, in that students living with two parents tended to play games more often than those living with one parent or in a different family arrangement. Additionally, students with siblings were more likely to play DGs than those without. Students were more likely to play computer games if they lived with a parent whose education was at a primary/secondary level (χ ^2^ = 7.340; *p* = 0.025) (Table 1). No significant differences were found in the distributions of demographic characteristics between early adolescents (aged 13–17) and late adolescents (aged 18–24) except for in paternal education, with late adolescents having less educated fathers than early adolescents (χ^2^ = 10.642; *p* = 0.005).

Participants reported higher mean gaming session durations (in minutes) during the weekends than during school days (148.1 ± 210.6 vs. 88.6 ± 120.3, respectively). Male students devoted more time to playing games than female students, both during school days (105.7 ± 134.4 (males) vs. 63.2 ± 90.7 (females); *z* = 3.369; *p* < 0.001) and free days (187.6 ± 238.8 (males) vs. 95.4 ± 151.6 (females); *z* = 6.294; *p* < 0.001).

The most common platforms for playing DGs were desktops/laptops (69.5%) and mobile phones (60.4%), with consoles (30.4%) chosen less often (Table 2). The male participants were significantly more likely than the female participants to choose the following devices to play games: desktop/laptop (82.2% vs. 55.2%; χ^2^ = 40.962; *p* < 0.001) and console (38.7% vs. 21.0%; χ^2^ = 17.698; *p* < 0.001) (Table 2). Males also played online games more often than females did (53.4% vs. 41.7%; χ^2^ = 6.458; *p* = 0.01).

### 3.2. Types of Games

The most commonly chosen game genre was strategy (57.1%), whereas war games (37.7%) were less popular (Table 3). All genres of games were selected significantly more often by male students than female students, except for educational games, which were more popular among female respondents (χ^2^ = 10.823; *p* < 0.001) (Table 3). Fighting and sports games were the most popular choices for male students, and education and strategy games were the most popular choices for female students (Table 3).

Analysis of playing time for various games in the study group during the week showed that the most time was devoted to war games. The comparison showed that a significantly longer game time was devoted to war games than for educational games (in minutes): 779.0 ± 945.6 vs. 404.9 ± 514.7 (*p* < 0.001), for adventure games: 779.0 ± 945.6 vs. 599.5 ± 812.3 (*p* < 0.001), and for races: 779.0 ± 945.6 vs. 565.0 ± 720.3 (*p* < 0.001). Statistically significant differences were also recorded in game time between fighting and educational games (in minutes): 722.6 ± 901.1 vs. 404.9 ± 514.7 (*p* < 0.001) and between fighting and sport games (in minutes): 722.6 ± 901.1 vs. 565.0 ± 720.3 (*p* < 0.001). Other differences between game time and selected type of games were not statistically significant (*p* > 0.05).

Almost all types of games were played more often by students who were poor sighted than by their blind peers. Educational games, however, were played slightly more often by students who were blind, but the difference was not significant. Almost all types of games were played more by respondents from urban areas than by those from rural areas, with significant associations identified for sports (χ^2^ = 4.984; *p* = 0.026), role playing (χ^2^ = 14.765; *p* < 0.001), and simulation games (χ^2^ = 4.861; *p* = 0.027) (Table 3). Family situation, the presence of siblings, and living at a boarding school had no significant impact on the choice of specific game genre. However, almost all types of games were selected less frequently by students who lived with two parents or at a boarding school than by their corresponding counterparts (Table 3).

### 3.3. Excessive Gaming

Over 12% of the students with VI took part in gaming sessions lasting 5 or more hours per day (school days mean time = 7 h 23 min, SD = 148 min; weekend mean time = 9 h 43 min, SD = 144 min); however, the male students were 5.5 times more likely to report excessive gaming than the female students (*p* = 0.000) (Table 4). In the study group, excessive gaming was observed almost twice as often among students with no siblings compared to those with siblings (*p* = 0.072), and over 1.5 times more often among students who had no parents compared with those who had both parents (*p* = 0.300) (Table 4).

## 4. Discussion

Our results have yielded important and novel information about DGs use among students with different levels of visual impairment. Our findings indicated that almost 84% of students with VI were users of DGs; however, poor-sighted participants selected all types of games more often than did those who were blind. Computer games are challenging for persons with VI because their use requires more attention resources, and their effectiveness in the game depends on the quality and efficiency of assistive computer software. Individuals with VI expect to obtain little useful information from pictures, even if they are clear and simple illustrations or high-contrast images with clear figures and slow movements. Blind persons are usually only occasional players of ordinary computer games, as these are mainly focused on visual output. Hence, future games would provide greater accessibility for blind users if they placed more emphasis on sound-based communication [32].

Our findings confirmed that students with VI devoted more time to playing DGs during the weekend than on school days. It has previously been noted that young persons with VI are more engaged in passive leisure activities than those without disabilities, and usually spend less time with their peers [33]. This may be attributed to the fact that young persons with VI are more prone to social isolation due to the limited opportunities for them to learn social skills by observing interactions in different contexts [34,35]. On the other hand, students with VI tend to be more engaged in peer contact through new media, thus compensating for the perceived traditional offline social isolation [19]; this could be reflected in the longer amounts of time spent playing games at the weekend.

Almost all types of games were selected less frequently by students with VI who lived with two parents or at a boarding school. Parents and school guardians of students with VI have an important impact on their children’s use of modern technology in all stages of development [4]. Conscious and controlled digital gaming behaviors require trust among family members, as well as support and communication between them, and positive guardianship might be an effective way to maintain healthy behaviors regarding the use of new technology among young persons with VI.

Our results also showed that all types of games were used more often by students from urban areas compared with those living in rural areas. Video game use may be greater in urban areas due to the relative lack of space available for outdoor activities and the lower level of safety [36]. Similarly, differences between rural and urban adolescents in Poland regarding their use of DGs seem to be related to differences in lifestyle and sedentary behavior [37]; similar differences have also been observed among adolescents in the US and Canada [38]. Additionally, gaming among rural adolescents may be limited by poorer access to internet and computers in nonurban areas [39].

Rideout et al. [40] reported that most game players were male, and that male gamers typically spent twice as much time playing as female gamers. Our study also confirmed that male students with VI played computer games significantly longer during school days and weekends than female students with VI. Male participants were also typified by a higher odds ratio than female participants with regard to excessive time spent gaming. Our findings demonstrate that males with VI, similar to male students with normal vision, are more in need of prevention strategies aimed at diversifying time spent on leisure activities and increasing behavioral control [41].

A gender gap was also found with regard to the selection of the genre of DGs, which is similar to that observed among young persons without disabilities [42,43]. In the present study, fighting and sports games tended to be chosen more often by male students, and education and strategy games were chosen more often by female students. According to a previous report [44], male gamers generally prefer games in which they can compete with other players, while female students are more likely to play logic and skill-training games.

Over 12% of individuals with VI in our study reported playing games for 5 or more hours per day; in contrast, a previous study found that only 9% of young gamers without disabilities played for the same time [16]. A higher incidence of comorbid mental disorders, such as depression and anxiety disorders, has been reported in individuals engaging in excessive gaming [45]. This may suggest that excessive gaming could have a negative impact on psychological functionality or serve as a negative coping strategy in dealing with mental health problems. Excessive gaming might occur in response to stressors such as social problems in the family, school life, or elsewhere [10].

Students with VI who had no parents or siblings were more likely to engage in excessive gaming than students living with two parents or having a brother or sister, respectively. This is consistent with a previous Polish study conducted with mainstream school students [46]. A greater risk of problematic gaming has been observed in young people who have a poor-quality relationship with their parents or those from single-parent [47] or blended families [48]. Gaming, in this sense, could reflect the desire of the adolescent to escape from the stress of a difficult family situation, or parents’ insufficient time and resources to support the child’s interests [49]. Parent–adolescent disclosure, known to facilitate positive social behavior, might be more challenging for incomplete families. Nonintrusive parental control could be a way of facilitating child disclosure, while a low level of nonintrusive parental control could contribute to lack of support and reduced social adjustment, which are known to be risk factors for developing problematic media use [50,51].

Our study has some limitations. First, the risk of potential problematic gaming was based solely on screen time devoted to DGs, and was not examined with regard to more complex gaming addiction measures. The reason for this was that although tools to assess problematic gaming are available in the literature [52,53], they are not adapted to the needs of persons with VI or are too bulky to be used by this population. We used the indicators of problematic gaming basing on 5 hours of daily play because it has been found to manifest negative effects close to addiction [16]. This particular choice for the study design seems reasonable, considering the existing controversies and disagreement on criteria for problematic media use [54]. Knowledge about the time spent on game playing was obtained based on the student’s declaration. It is recommended to use independent measurement methods (recording the start and end times of the game with a counter) to receive more precise results in this context.

In addition, personal preferences regarding specific game types were not measured, and it was thus not clear whether this choice was based on personal preference or the availability of games indicated in the questionnaire. However, this limitation could not be overestimated in the current study, as it was addressed in several ways. A relatively large sample was recruited to minimize confounding factors, and access to DG types was diversified by ensuring the heterogeneity of our sample, which was selected from different communities and regions of Poland. The prevalence of electronic devices used by students with VI was also collected. Unfortunately, we did not specify what kind of game formats and platforms were chosen. It is worthwhile to take this into account in the future development of qualitative studies. Finally, the study was conducted before the pandemic and isolation at home, but it presents indications for game development in a post-COVID time when the digitalization of society is now growing.

## 5. Conclusions

The current survey indicated a tendency towards excessive gaming in a significant proportion of young persons with VI. Sociodemographic variables are important in predicting gaming prevalence or screen time, but further research focused on establishing possible mediators such as parental attitudes towards DGs are necessary for identifying problematic gaming behaviors among students with VI. Moreover, parents and healthcare providers play an important role in the early intervention on excessive playing by modulation and controlled use of DGs by their charges [55]. The Coronavirus pandemic surely changed the way of thinking about time spent in front of the computer, internet usage and online gaming. People are housebound, and wear masks when outside, which affects social interactions. Gaming can help to relief stress, but also brings a risk of addiction [56]. Our study shows the importance of relationships with caregivers, and in this new reality—with reality more concentrated on online functionality—it seems to be even more important. It seems that psychoeducational training both for caregivers and adolescents would be needed in order to learn how to recognize risky behaviors, where to seek help, and how to develop more adaptive strategies for coping with difficulties. An interesting field of further investigation would also be the psychological features connected with addiction within a group of students with VI.

Our study also confirmed that a wide range of the study group are regular gamers, but the blind students were only a small percentage. Game designers claim that playability is the most important determinant during the adaptation of a game to the ergonomic characteristics and needs of people with disabilities. Hence, our results shows the need to adjust commercial games for gamers with VI to provide them equal access to information and communication technologies Designers should develop and adapt rich, immersive, and engaging gaming environments to the blind by including easier levels of challenge for those playing with degraded vision [57]. It is recommended to provide an appropriate level of playability among blind people by an alternative representation of a graphical user interface, such as audio and tactile computer games, or as a combination of the two basic models.

Applying game mechanics to non-game contexts in order to engage audiences and generate motivational and cognitive benefits is a current trend in therapy and rehabilitation [58]. Our research confirmed the students’ interest in computer games, especially in strategic games, which are most often played on computers and mobile phones. In response to the needs of adolescents with VI, it is worth taking these results into account in planning future preventive actions based on gamifications in various areas of life.

## Figures and Tables

**Table 1:** Sociodemographic variables of the study group.

Sociodemographic Variables	Total (*n* = 490; 100.0%)		Gamers in the Study Group (*n* = 410; 83.7%)	
	*N*	%	*N*	%
Age group				
13–17	241	49.2	202	49.3
18–24	249	50.8	208	50.7
Sex				
Male students	259	52.9	234	57.1
Female students	231	47.1	176	42.9
Place of residence				
Urban	319	65.1	275	67.1
Rural	171	34.9	135	32.9
Family situation				
Living with two parents	331	68.5	274	67.8
Living with one parents	98	20.3	83	20.5
Other situation	54	11.2	47	11.7
Boarding school				
Yes	271	55.5	223	54.7
No	217	44.5	185	45.3
Sibling				
Yes	412	86.6	343	86.2
No	64	13.4	55	13.8
Level education of mother				
Primary/secondary	253	57.3	209	56.8
Post-Secondary	137	31.1	114	31.0
Higher	51	11.6	45	12.2
Level education of father				
Primary/secondary	257	62.7	212	61.3
Post-Secondary	106	25.8	88	25.4
Higher	47	11.5	46	13.3

**Table 2:** The prevalence of electronic devices used by students with visual impairment (in percentages).

	Students with VI	Male Students	Female Students
Desktop/laptop	69.5	82.2	55.2 ^a^
Tablet	38.1	34.1	42.4
Mobile phone	60.4	63.4	56.7
Console	30.4	38.7	21.0 ^b^

Note: Chi-square calculations for the independence of gender and use of electronic devices: ^a^ chi^2^ = 40.962; *p* < 0.001; ^b^ chi^2^ = 17.698; *p* = 0.001; VI: Visual Impairment.

**Table 3:** Prevalence of specific game genres, including sociodemographic data (in percentages).

Sociodemographic Variables	Strategy	Fighting	Sports	Education Puzzles	Action/ Adventure	War	Role-Playing Game	Simulation
Total								
	57.1	44.4	53.4	41.4	50.6	37.7	46.5	45.0
Age group (years)								
13–17	53.7	46.1	50.4	35.5	46.3	38.1	47.6	43.0
18–24	60.3	42.8	56.2	47.1 ^a^	54.8	37.3	45.4	46.9
^a^ chi^2^ = 6.564; *p* = 0.01								
Sex								
Male students	65.7	68.1	67.1	34.4	56.2	62.1	60.8	54.9
Female students	47.5 ^a^	17.6 ^b^	38.0 ^c^	49.3 ^d^	44.3 ^e^	9.9 ^f^	30.6 ^g^	34.1 ^h^
^a^ chi^2^ = 15.831; *p* < 0.001; ^b^ chi^2^ = 121.99; *p* < 0.001; ^c^ chi^2^ = 40.398; *p* < 0.001; ^d^ chi^2^ = 10.823; *p* < 0.001; ^e^ chi^2^ = 6.612; *p* = 0.01 *p* = 0.010; ^f^ chi^2^ = 136.916; *p* < 0.001; ^g^ chi^2^ = 42.662; *p* < 0.001; ^h^ chi^2^ = 20.443; *p* < 0.001								
Level of VI								
Blind	53.6	26.1	31.4	44.3	38.6	20.0	35.7	21.4
Poor-sighted	57.8	47.5 ^a^	57.1 ^b^	40.9	52.8 ^c^	40.7 ^d^	48.4	49.1 ^e^
^a^ chi^2^ = 10.972; *p* = 0.001; ^b^ chi^2^ = 15.863; *p* < 0.001; ^c^ chi^2^ = 4.791; *p* = 0.029; ^d^ chi^2^ = 10.933; *p* = 0.001; ^e^ chi^2^ = 18.437; *p* < 0.001								
Place of residence								
Urban	58.8	46.8	57.1	40.9	51.8	40.7	53.0	48.7
Rural	54.0	40.0	46.4 ^a^	42.4	48.5	32.1	34.4 ^b^	38.0 ^c^
^a^ chi^2^ = 4.984; *p* = 0.026; ^b^ chi^2^ = 14.765; *p* < 0.001; ^c^ chi^2^ = 4.861; *p* = 0.027								
Family situation								
Living with two parents	55.2	42.8	50.0	41.3	49.2	35.5	44.9	42.4
Living with one parent	59.6	46.8	60.9	43.1	52.3	46.4	50.5	50.0
Other situation	62.2	46.0	54.1	37.8	59.5	35.1	57.1	52.8
Boarding school								
Yes	57.4	41.7	50.4	43.2	48.7	34.2	41.6	42.4
No	56.5	47.6	56.5	38.7	53.1	41.8	52.4 ^a^	47.6
^a^ chi^2^ = 5.360; *p* = 0.021								
Siblings								
Yes	57.4	43.8	52.0	41.3	51.6	37.1	46.4	44.3
No	53.5	43.3	57.4	45.0	44.8	36.7	50.0	50.8

**Table 4:** Respondents’ participation in gaming sessions lasting 5 or more hours according to type of visual impairment and sociodemographic variables.

Sociodemographic Variables	Total				Boys				Girls			
	*n* (%)	OR	95% CI	*p*	*n* (%)	OR	95% CI	*p*	*n* (%)	OR	95% CI	*p*
Age group												
15–17	28 (47.5)	1.0			23 (46.9)	1.0			5 (50.0)	1.0		
18–24	31 (52.5)	1.19	0.67–2.08	0.552	26 (53.1)	1.17	0.63–2.20	0.613	5 (50.0)	0.92	0.26–3.29	0.900
Sex												
Male	49 (83.1)	5.56	2.55–12.1	0.000	-	-	-	-	-	-	-	-
Female	10 (16.9)	1.0			-	-	-	-	-	-	-	-
Type of VI												
Blind	8 (13.6)	1.0			6 (12.2)	1.0			2 (20.0)	1.0		
Low vision	51 (86.4)	1.06	0.43–2.56	0.904	43 (87.8)	1.58	0.63–4.01	0.330	8 (80.0)	0.49	0.10–2.45	0.380
Place of residence												
Urban	42 (71.2)	1.44	0.77–2.71	0.254	35 (71.4)	1.28	0.64–2.54	0.483	7 (70.0)	1.40	0.35–5.62	0.630
Rural	17 (28.8)	1.0			14 (28.6)	1.0			3 (30.0)	1.0		
Family situation												
Living with two parents	41 (70.7)	1.0			35 (72.9)	1.0			6 (60.0)	1.0		
Living with one parent	10 (17.2)	0.69	0.33–1.44	0.316	7 (12.5)	0.57	0.24–1.37	0.208	3 (30.0)	1.37	0.33–5.72	0.664
Other situation	7 (12.1)	1.61	0.65–3.96	0.300	6 (14.6)	2.04	0.71–5.84	0.181	1 (10.0)	1.30	0.15–11.5	0.814
Boarding school												
Yes	33 (55.9)	0.99	0.59–1.65	0.961	28 (42.9)	1.12	0.60–2.11	0.722	5 (50.0)	0.77	0.21–2.75	0.683
No	26 (44.1)	1.0			21 (57.1)	1.0			5 (50.0)	1.0		
Sibling												
Yes	46 (79.3)	1.0			38 (79.2)	1.0			8 (80.0)	1.0		
No	12 (20.7)	1.92	0.94–3.93	0.072	10 (20.8)	1.99	0.88–4.50	0.098	2 (20.0)	0.68	0.14–3.36	0.632

CI: confidence interval; OR: odds ratio.

## Data Availability

The data presented in this study are available on request from the corresponding author.
